# Prize Essay

**Published:** 1853-04

**Authors:** 


*Prize Essay.*—The prize which we offered in our October number for the best article on Odontalgia, has been awarded to Dr. Togg. The article will be published in the July number of the Journal.

